# Implementation of the Treat All Policy Among Persons with HIV Infection Enrolled in Care But Not on Antiretroviral Therapy — India, May 2017–June 2018

**DOI:** 10.15585/mmwr.mm6747a2

**Published:** 2018-11-30

**Authors:** Kiren Mitruka, Manish Bamrotiya, Reshu Agarwal, Anwar Parvez, Ramesh Reddy Allam, Srilatha Sivalenka, Pramod Deoraj, Rajendra Prasad, Uma Devi, Padmaja Keskar, Shrikala Acharya, Priya Kannan, Ramesam Ganti, Malay Shah, Shashikant Todmal, Praveen Kumar, Nalini Chava, Ajit Rao, Sukarma Tanwar, Melissa Nyendak, Tedd Ellerbrock, Timothy H. Holtz, R.S. Gupta

**Affiliations:** ^1^Division of Global HIV and TB, Center for Global Health, CDC; ^2^National AIDS Control Organization, Government of India; ^3^Division of Global HIV and TB, CDC India; ^4^UW International Training and Education Center for Health, Private Limited, New Delhi, India; ^5^SHARE INDIA, Telangana, India; ^6^Maharashtra State AIDS Control Society, Mumbai, India; ^7^Andhra Pradesh State AIDS Control Society, Vijayawada Rural City, India; ^8^Mumbai District AIDS Control Society, Mumbai, India.

Since September 2015, the World Health Organization has recommended antiretroviral therapy (ART) for all persons with human immunodeficiency virus (HIV) infection, regardless of clinical stage or CD4 count (*1*). This Treat All policy was based on evidence that ART initiation early in HIV infection as opposed to waiting for the CD4 count to decline to certain levels (e.g., <500 cells/mm^3^, per previous guidelines), was associated with reduced morbidity, mortality, and HIV transmission (*2*–*4*). Further, approximately half of persons enrolled in non-ART care that included monitoring for HIV disease progression (i.e., in pre-ART care) were lost to follow-up before becoming ART-eligible (*5*). India, the country with the third largest number of persons with HIV infection in the world (2.1 million), adopted the Treat All policy on April 28, 2017. This report describes implementation of Treat All during May 2017–June 2018, by India’s National AIDS Control Organization (NACO) and partners, by facilitating ART initiation among persons previously in pre-ART care at 46 ART centers supported by the U.S. President’s Emergency Plan for AIDS Relief (PEPFAR)* in six districts in the states of Maharashtra and Andhra Pradesh. Partners supported these 46 ART centers in identifying and attempting to contact persons who were enrolled in pre-ART care during January 2014–April 2017, and educating those reached about Treat All. ART center–based records were used to monitor implementation indicators, including ART initiation. A total of 9,898 (39.6%) of 25,007 persons previously enrolled in pre-ART care initiated ART; among these 9,898 persons, 6,315 (63.8%) initiated ART after being reached during May 2017–June 2018, including 1,635 (16.5%) who had been lost to follow-up before ART initiation. NACO scaled up efforts nationwide to build ART centers’ capacity to implement Treat All. Active tracking and tracing of persons with HIV infection enrolled in care but not on ART, combined with education about the benefits of early HIV treatment, can facilitate ART initiation.

Among the estimated 2.1 million persons with HIV infection in India in 2017,^†^ 1.7 million (81%) had received a diagnosis, and 1.2 million receive free ART. Through PEPFAR, CDC and its implementing partners^§^ provide technical support to improve HIV care and treatment in three districts each in Maharashtra (32 ART centers) and Andhra Pradesh (14), the two states with the highest prevalence of HIV infection. In 2015, these six districts accounted for 36% and 39% of persons with HIV infection in Maharashtra (301,453) and Andhra Pradesh (including Telangana, which has since separated from Andhra Pradesh) (394,661), respectively (*6*).

On May 1, 2017, state and district health authorities, in collaboration with CDC and implementing partners, began activities to implement Treat All by projecting antiretroviral needs through estimates of persons who were alive and on ART and the assumption that 50% of enrolled persons not on ART would initiate ART within 6 months. CD4 laboratory registers and electronic databases at ART centers were used to identify persons with HIV infection enrolled at one of the 46 PEPFAR-supported ART sites in Maharashtra or Andhra Pradesh who had a CD4 count or clinic visit during January 2014–April 2017, but who were not on ART (i.e., in pre-ART care before Treat All). Persons who had died, transferred out, opted out, or started ART were excluded. Paper records were reviewed to deduplicate entries with matching names and addresses and to verify ART status and contact information. Persons in pre-ART care before Treat All implementation were categorized as 1) in active care (having had a CD4 count or clinical assessment every 6 months) or 2) lost to follow-up (not having been seen at the center for ≥12 months) (*7*).

CDC and implementing partners supported ART centers to scale up and systematize NACO-recommended activities for tracking and tracing persons with HIV infection in pre-ART care. ART center counselors made three attempts to contact each person previously in pre-ART care by telephone and used standardized materials describing the benefits of early ART to educate those who were reached. Home visits were scheduled for those persons who were not reached by telephone, who declined to go to the ART center, or who agreed to go but did not. Persons who missed appointments before or after ART initiation were contacted by telephone within approximately 7 and 2 days, respectively. Partners adapted existing tracking tools to monitor missed appointments, ART initiation, and retention on ART, defined as documented receipt of ART at specific time points (e.g., 6 or 12 months); implementation indicator data, including ART initiation and retention, were entered and maintained in electronic spreadsheets.

Among 25,007 persons in pre-ART care, counselors reached 12,691 (50.7%); among those reached, 1,950 (15.4%) reported already having initiated ART since May 1, 2017 (Table). Among the remaining 10,741 persons reportedly not on ART, 10,243 (95.4%) agreed to visit the ART center, 6,524 (63.7%) of whom did visit the center before June 30 or within 2 weeks of the appointment (whichever period was longer). Among these 6,524 persons, 6,315 (96.8%) initiated ART. Among 6,564 persons previously in pre-ART active care who agreed to visit the center, 4,836 (73.7%) visited the ART center, compared with 1,688 (45.9%) of 3,679 persons who had been lost to follow-up. Nearly all (97.0%) persons in both groups who visited centers initiated ART.

**TABLE:** Follow-up of persons with human immunodeficiency virus (HIV) infection enrolled in care but not on antiretroviral therapy (pre-ART) who were contacted during implementation of the Treat All policy at 46 ART centers,* by pre-ART care status — Maharashtra and Andhra Pradesh states, India, May 2017–June 2018

Contact efforts/Outcome	Pre-ART HIV care status no. (%)		
	Total	Active care^†^	Lost to follow-up^§^
**Contact attempted**	**25,007 (100.0)**	13,308 (100.0)	11,699 (100.0)
**Reached**	**12,691 (50.7)**	8,139 (61.2)	4,552 (38.9)
By telephone	**9,441 (74.4)**	6,508 (80.0)	2,933 (64.4)
By home visit	**3,250 (25.6)**	1,631 (20.0)	1,619 (35.6)
**Already on ART (% of persons reached)^¶^**	**1,950 (15.4)**	1,368 (16.8)	582 (12.8)
**Not on ART (% of persons reached)**	10,741 (84.6)**	6,771 (83.2)	3,970 (87.2)
**Agreed to visit ART center (% of persons not on ART)**	**10,243 (95.4)**	6,564 (96.9)	3,679 (92.7)
**Visited ART center (% of persons who agreed to visit)**	**6,524 (63.7)**	4,836 (73.7)	1,688 (45.9)
**Initiated ART (% of persons who visited ART center)**	**6,315 (96.8)**	4,680 (96.8)	1,635 (96.9)

**Abbreviation: **AIDS = acquired immunodeficiency syndrome.

* Supported by U.S. President’s Emergency Plan for AIDS Relief.

^†^ Defined as having had a CD4 count or clinical visit during May 2016–April 2017.

^§^ Defined as having had a CD4 count or clinical visit during January 2014–April 2016, and subsequently did not visit the ART center for ≥12 months.

^¶^ Among 12,691 persons reached, 1,950 (15.4%) reported having already visited the ART center and initiating ART since May 1, 2017.

** Three of 10,741 persons who reported not being on ART were eventually identified through medical records to be on ART.

The median interval from the agreement to visit the center to the actual visit was 18 days (interquartile range [IQR] = 4–61 days) and from visiting the center until ART initiation was 3 days (IQR = 1–9 days). Among 21,631 (86%) persons previously in pre-ART care with available CD4 data, the median CD4 count was 571 cells/mm^3^ (IQR = 412–759); among the 6,524 persons who visited the ART center, the median CD4 count was 645 cells/mm^3^ (IQR, 522–826).

In addition to the 6,315 persons who initiated ART after being reached, 3,583 persons were found to have already initiated ART after the May 1 implementation of Treat All; 1,950 were identified through outreach, and 1,633 were identified while monitoring center records for visits. Thus, among all 25,007 persons with HIV infection previously in pre-ART care, 9,898 (39.6%) persons initiated ART during May 2017–June 2018. Among 6,315 persons who began ART after being reached, 4,463 of 5,247 (85.1%) were retained in care at 6 months and 682 of 809 (84.3%) at 12 months.

Before implementation of Treat All, a median of 1,847 (IQR = 1,615–2,007) persons with HIV infection initiated ART each month at the 46 ART centers. After May 1, 2017, this number increased, peaking at 3,797 in July, at which time persons previously in pre-ART care accounted for approximately half (52%) of all ART initiations (Figure); thereafter, this proportion declined to approximately 2%. During the course of the 14-month implementation of Treat All, ART center staff members required a decreasing level of support from implementing partners in responding to questions about Treat All, tracking and tracing activities, and data management.

**FIGURE:**
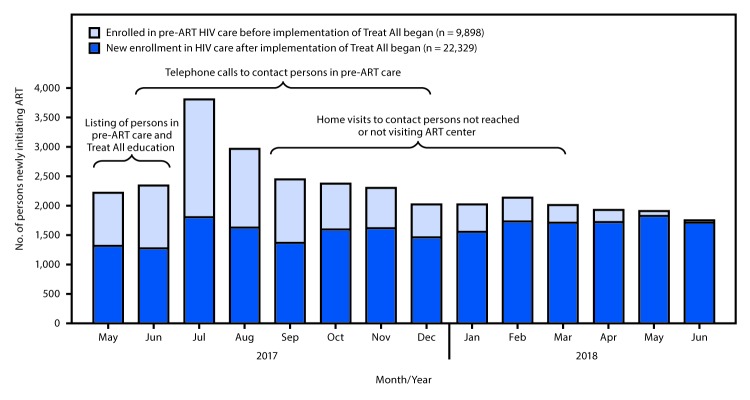
Number of persons with human immunodeficiency virus (HIV) infection newly initiating antiretroviral therapy (ART) (N = 32,227) among those who enrolled in care before* or after implementation of the Treat All policy† at 46 centers supported by the President’s Emergency Plan for AIDS Relief (PEPFAR), by month and year — Maharashtra and Andhra Pradesh states, India, May 1, 2017‒June 30, 2018 **Abbreviation:** AIDS = acquired immunodeficiency syndrome. * Pre-ART; persons enrolled in non-ART HIV care. ^†^ The Treat All policy, based on evidence that ART initiation early in HIV infection is associated with reduced morbidity, mortality, and HIV transmission, was adopted by India on April 28, 2017, and policy implementation began on May 1, 2017.

## Discussion

This is the first report describing a national ART program’s effort to facilitate ART initiation among persons with HIV infection enrolled in pre-ART care immediately after adoption of the Treat All policy. During the third and fourth months of implementation at the 46 PEPFAR-supported ART centers in India, the number of persons previously in pre-ART care returning to ART centers resulted in a doubling of the median number of persons initiating ART each month. Approximately two thirds of those who initiated ART within 14 months of implementation of Treat All did so after active follow-up. This effort to facilitate ART initiation improved ART centers’ capacity in counseling, tracking and tracing, and managing data, prompting NACO to scale up activities to implement Treat All nationwide. Ensuring linkage to ART is an important factor in realizing population-level benefits of the Treat All policy through reducing HIV transmission (*8*).

Approximately half of all persons previously in pre-ART care were reached by telephone and home visits, highlighting the importance of regularly updating contact information. Although most persons who were reached and not on ART did agree to visit an ART center, fewer than two thirds actually did so. The high median CD4 count among persons previously in pre-ART care who were reached suggests that many were likely asymptomatic. Thus, education about the Treat All policy is needed to address the misperception, based on earlier guidance, that this population is not eligible for ART. Most persons previously in pre-ART care (97%) who visited ART centers initiated ART within a median of 3 days; early data determined a 12-month ART retention of 84%, which is 13 percentage points higher than the national average of 71% (*9*).

The findings in this report are subject to at least four limitations. First, ART center–based records might be subject to data entry errors. Second, persons who initiated ART at other centers might have been missed. Third, because verified, deduplicated data on ART status were unavailable for persons previously enrolled in pre-ART care at the national level, the trend in new ART initiations could not be assessed in districts not supported by PEPFAR. Finally, direct causality cannot be inferred from the activities described in this report and the observed trend in ART initiations.

With half of persons in pre-ART care not yet reached, the eventual decline of new ART initiations to levels similar to those before adoption of Treat All suggests the need for ongoing education about the policy. Continued efforts also are needed to reach persons with HIV infection who are not on ART to understand and address barriers to ART initiation. Further, the full individual and public health benefits of Treat All can only be realized by overcoming program challenges for early HIV diagnosis and linkage to ART, rapid ART initiation, and support of ART adherence and retention among all persons with HIV infection (*10*). India is actively working to improve each of these areas through efforts that include implementation of patient-centered service delivery models to maximize the number of persons with HIV infection receiving ART and to improve quality of care.

**Summary**

**What is already known about this topic?**

The World Health Organization’s Treat All policy recommends antiretroviral therapy (ART) for all persons with human immunodeficiency virus (HIV) infection immediately after HIV diagnosis.

**What is added by this report?**

To implement Treat All in India, 46 ART centers in two states supported by the President’s Emergency Plan for AIDS relief attempted to contact 25,007 persons enrolled in HIV care but not receiving ART; 9,898 (40%) subsequently initiated ART over a 14-month period. Among those initiating ART, 6,315 (64%) began ART after being reached, including 1,635 (17%) who had been lost to follow-up.

**What are the implications for public health practice?**

Tracking and tracing and education about benefits of early HIV treatment among persons with HIV infection who are not on ART can facilitate implementation of Treat All in India.

## Footnotes

* https://www.pepfar.gov/about/270968.htm.

† https://nhp.gov.in/nhpfiles/hivaidsact.pdf.

§ CDC’s implementing partners for HIV Care and Treatment are UW International Training and Education Center for Health and SHARE INDIA.
